# Analysis of the improvement of serological indexes in patients with diabetic nephropathy and hypertension using Valsartan combined with Nifedipine controlled-release regimen

**DOI:** 10.12669/pjms.38.6.5871

**Published:** 2022

**Authors:** Lili Cai, Haili Zhu

**Affiliations:** 1 Lili Cai, Department of Nephrology, Huzhou Traditional Chinese Medicine Hospital, Affiliated to Zhejiang Chinese Medical University, Huzhou, Zhejiang Province 313000, P.R. China; 2 Haili Zhu, Department of Nephrology, Lianshi people’s Hospital of Nanxun District, Huzhou, Zhejiang Province 313000, P.R. China

**Keywords:** Diabetic nephropathy, Hypertension, Valsartan, Nifedipine controlled release tablets, Renal function, Blood pressure

## Abstract

**Objective:**

To investigate the effect of valsartan combined with nifedipine controlled-release Tablets on diabetic nephropathy (DN) patients with hypertension.

**Methods:**

The clinical records of 80 DN patients with hypertension registered in our hospital from April 2020 to April 2021 were collected. The records showed that 38 patients were treated with oral nifedipine controlled-release tablets (control group) and 42 - with oral valsartan combined with nifedipine controlled-release tablets (observation group). The improvement of serological indexes after treatment was compared and analyzed between the two groups.

**Results:**

After treatment, the levels of fasting blood glucose (FBG), systolic and diastolic blood pressure, bone oligomeric matrix protein (COMP), thrombin regulatory protein (TM) and Microalbumin (mALB) in the observation group were lower than those in the control group (P<0.05), while the level of angiopoietin-1 (Ang-1) was higher than those in the control group (P<0.05). After the treatment, the levels of homocysteine (Hcy), Cystatin C (CysC) and transforming growth factor β1(TGF-β1) in the observation group were lower than those in the control group (P<0.05). The levels of adiponectin (APN), aldosterone (ALD) and gastric growth promoting factor (ghrelin) in the observation group after the treatment were lower than those in the control group (P<0.05).

**Conclusions:**

A combination of valsartan and nifedipine controlled-release tablets in DN patients with hypertension can effectively control blood glucose and blood pressure, improve the serological indexes such as COMP, TM, mAlb, Ang-1, Hcy, CysC, TGF-β1, APN, ALD and ghrelin, and potentially reduce and delay renal function damage.

## INTRODUCTION

Diabetic nephropathy (DN) is a common complication of diabetic microangiopathy, and is responsible for 44% cases of end-stage renal disease.^1^ The pathological changes of DN are mainly glomerular hypertrophy, mesangial widening, basement membrane thickening and increased permeability.^2^ As the disease progresses, patients may develop proteinuria, accompanied by hypertension, serious damage to renal function and other symptoms. In addition, many clinical studies have shown that hypertension is the main factor causing and promoting the progress of DN. The increase of arterial blood pressure can increase the internal pressure of glomerulus, aggravate the proteinuria, accelerate the deterioration of renal function and increase the risk of cardiovascular disease.^3^

For the treatment of such patients, in addition to the symptomatic treatment, strict control of hypertension should be carried out, and the target blood pressure should be lower than that of non-diabetic patients (125/ 75mmHg as compared to 130/ 80mmHg, respectively).^4^ Valsartan and nifedipine controlled-release tablets are common drugs for the treatment of hypertension. Valsartan is an angiotensin II receptor blocker, which can inhibit vasoconstriction and reduce blood pressure by selectively binding to angiotensin 1 (AT1) receptor. Nifedipine controlled-release tablets are a kind of calcium antagonist, which can promote vasodilation and effectively control blood pressure by reducing the concentration of calcium in blood vessels.^5^,^6^ In recent years, valsartan combined with Nifedipine controlled-release tablets have been selected to treat DN patients with hypertension, with increased curative effect.

In this study, serum indicators of diabetic nephropathy such as COMP, TM, mALB, Ang-1, Hcy, CysC and TNF-β1 were used as efficacy evaluation indicators of the clinical effect of valsartan combined with nifedipine controlled release tablets in patients with DN and hypertension.

## METHODS

Clinical records of DN patients with hypertension, treated in our hospital from April 2020 to April 2021, were collected for retrospective analysis was performed. A total of 80 patients were included in the study (48 males and 32 females). Records indicated that 38 patients (control group) were treated with nifedipine controlled-release tablets (Bayer Medical and Health Care Co., Ltd, J20180025, 30g/time, once a day, lasting for six months), and 42 patients (observation group) were treated with a combination of valsartan (Beijing Novartis Pharmaceutical Co., Ltd., H20040217, 80mg / time, once a day for six months) and Nifedipine controlled-release tablets (30g / time, once a day, lasting for six months).

### DN diagnostic criteria were as follows:^7^

• The history of Type-II diabetes for more than 6~10 years.
• Clear manifestations and indications of renal dysfunction such as proteinuria, edema and hypoproteinemia;
• Diabetic retinopathy;
• Stromal hyperplasia and basement membrane thickening in glomerular mesangium, and glassy lesions in the wall of vascular arterioles visible during pathological examination;
• Pathological examination (renal biopsy) showed stromal hyperplasia and basement membrane thickening in the mesangium of glomerulus, and glassy lesions in the wall of arterioles of blood vessels.

### Diagnostic criteria for hypertension:^8^

The mean value of three blood pressure measurements on different days (without medication), systolic blood pressure ≥ 140mmHg, and / or diastolic blood pressure ≥ 90mmHg.

### Inclusion criteria:

• Meets the above diagnostic criteria of DN and hypertension at the same time;
• Complete medical records;
• Good compliance during treatment;
• No mental and neurological abnormalities.

### Exclusion criteria:

• Severe dysfunction of other organs;
• Diabetic ketoacidosis.
• Urinary tract diseases;
• Contraindications to this study;
• Pregnant and lactating women;
• Lost for follow-up.

### Ethical approval

The medical ethics Committee of our hospital had approved this study (Approval number: 202104011, Date: 2021-04-11).

All the medical records included basic information of patients and the related indexes, collected on the date of admission, three months after treatment and six months after treatment. Levels of FBG, systolic blood pressure and diastolic blood pressure were measured. Levels of renal vascular-related indexes COMP, Ang-1 and TM were detected by enzyme-linked immunosorbent assay kits (Shanghai Xitang Biotechnology Co., Ltd).The level of mALB was detected by immunoturbidimetry assay kits(Shanghai Xitang Biotechnology Co., Ltd). Levels of Hcy, CysC and TGF-β1 were measured using automatic biochemical analyzer (Meirui, bs-280). Levels of APN, ALD and ghrelin were detected by respective enzyme-linked immunosorbent assay kits (Wuhan bode Biotechnology Co., Ltd).

### Statistical Analysis

**S**PSS 22.0 was used for data processing. T-test is performed, with [n (%)] representing non grade count data, and the test method is *x*^2^; (*x̅*± s) representing the measurement data. When p<0.05, the difference was considered statistically significant.

## RESULTS

Records of a total of 80 patients met the inclusion criteria of this retrospective study. The control group (n=38) included 22 males and 16 females, with the mean age of (55.26±8.11) years, the course of DN in this group was (8.44±1.72) years. The observation group (n=42) included 24 males and 18 females, with the average age was (56.83±7.58) years, and the average course of DN of (8.59±1.46) years. There was no significant difference between the two groups in gender, age and DN course (P>0.05). The changes of blood glucose and blood pressure before and after the treatment are shown in Table-I. There was no significant difference in FBG, systolic and diastolic blood pressure between the two groups before treatment (P>0.05). At three and six months after the treatment, FBG, systolic blood pressure and diastolic blood pressure decreased in both groups and were significantly lower in the observation group as compared to the control (P<0.05). Before treatment, there was no significant difference in COMP, Ang-1, TM and mALB levels between the two groups (P>0.05). At three and six months after treatment, the levels of COMP, TM and mALB in the two groups decreased compared with those before treatment. After the treatment, levels of Ang-1 were lower (P<0.05) in the observation group as compared to the control group, while levels of mALB in the observation group were higher (P<0.05), as shown in Table-II. Before treatment, Hcy, CysC and TGF- β levels were similar in both groups (P>0.05). At three and six months after treatment, the levels of Hcy, CysC and TGF-β1 in the two groups were lower than those before treatment. Moreover, after the treatment patients in the observation group had lower Hcy, CysC and TGF-β1 than patients in the control group (P<0.05), as shown in Table-III. Before treatment, there was no significant difference in the levels of APN, ALD and ghrelin between the two groups (P>0.05). At three and six months after treatment, the levels of APN in the two groups were higher than those before treatment, and the levels of ALD and ghrelin in the observation group were lower than those of the control group (P<0.05; Table-IV).

**Table I:** Comparison of blood glucose and blood pressure levels between the two groups before and after treatment (*x̅*±s).

Group(n)	FBG (mmol/L)			Systolic blood pressure (mmHg)			Diastolic blood pressure (mmHg)		

	before therapy	3 months after treatment	6 months after treatment	before therapy	3 months after treatment	6 months after treatment	before therapy	3 months after treatment	6 months after treatment
Control group (n=38)	10.37±2.23	8.42±2.01^*^	6.44±1.83^*^	155.97±6.55	135.74±5.20^*^	130.44±4.87^*^	98.84±4.34	90.66±3.51^*^	85.36±3.15^*^
Observation group(n=42)	10.26±2.13	6.57±1.80^*^	5.52±1.65^*^	154.90±6.83	131.14±5.75^*^	125.78±5.32^*^	98.33±3.71	86.45±3.33^*^	76.33±3.23^*^
t	0.218	4.348	2.372	0.712	3.733	4.071	0.565	5.497	12.631
P	0.828	<0.001	0.020	0.478	<0.001	<0.001	0.574	<0.001	<0.001

***Note***: Compared with this group before treatment

* p<0.05.

**Table II:** Comparison of related indexes of renal blood vessels before and after treatment in the two groups (*x̅*±s).

Group (n)	Time	COMP (ng/ml)	Ang-1 (ng/L)	TM (mg/L)	mALB (mg/L)
Control group (n=38)	before therapy	83.92±3.46	11.92±2.53	73.78±5.65	64.92±5.80
	3 months after treatment	72.92±3.11^*^	15.21±2.78^*^	67.31±5.47^*^	57.78±5.18^*^
	6 months after treatment	65.60±2.87^*^	17.34±2.99^*^	63.50±4.68^*^	51.65±4.37^*^
Observation group (n=42)	before therapy	84.14±3.70	12.16±2.47	74.28±5.63	65.14±6.45
	3 months after treatment	66.64±3.02^*#^	17.54±2.60^*^	61.86±5.30^*^	50.11±5.72^*^
	6 months after treatment	54.54±2.84^*#^	20.73±2.68^*#^	53.93±4.81^*#^	38.21±4.47^*#^

***Note***: Compared with this group before treatment

* P<0.05<Compared with the control group at the same time

# p<0.05.

**Table III:** Comparison of Hcy, CysC and TGF-β1 levels before and after treatment in the two groups (*x̅*±s).

Group (n)	Hcy (umol/L)			CysC (mg/L)			TGF-β1 (mmHg)(mg/L)		

	before therapy	3 months after treatment	6 months after treatment	before therapy	3 months after treatment	6 months after treatment	before therapy	3 months after treatment	6 months after treatment
Control group (n=38)	12.44±2.20	9.81±2.07^*^	7.97±1.81^*^	0.99±0.27	0.86±0.23^*^	0.66±0.19^*^	157.36±6.67	142.44±5.57^*^	132.36±4.86^*^
Observation group (n=42)	12.68±2.10	8.15±2.05^*^	6.78±1.69^*^	0.95±0.27	0.73±0.24^*^	0.55±0.20^*^	158.07±5.84	134.04±5.84^*^	118.26±5.03^*^
t	0.486	3.592	3.042	0.653	2.384	2.661	0.482	6.557	12.710
P	0.529	0.001	0.003	0.515	0.020	0.009	0.631	<0.001	<0.001

***Note***: Compared with this group before treatment

* p<0.05.

**Table IV:** Comparison of APN, ALD, and ghrelin levels before and after treatment in the two groups (*x̅*±s).

Group (n)	APN (mg/L)			ALD (pg/ml)			Ghrelin (ug/L)		

	before therapy	3 months after treatment	6 months after treatment	before therapy	3 months after treatment	6 months after treatment	before therapy	3 months after treatment	6 months after treatment
Control group(n=38)	5.88±1.26	7.60±1.64^*^	10.08±1.73^*^	151.87±6.52	140.71±5.16^*^	135.47±3.69^*^	14.53±2.12	12.24±1.99^*^	8.46±1.76^*^
Observation group (n=42)	5.82±1.14	9.09±1.38^*^	12.44±1.58^*^	152.04±6.62	134.64±6.05^*^	118.66±4.91^*^	14.69±2.45	10.70±2.03^*^	6.52±1.83^*^
t	0.253	4.420	6.348	0.122	4.797	17.137	0.318	3.405	4.804
P	0.801	<0.001	<0.001	0.903	<0.001	<0.001	0.751	0.001	<0.001

***Note***: Compared with this group before treatment

* p<0.05.

## DISCUSSION

The results of this study show that valsartan combined with nifedipine controlled-release tablets can effectively improve the relevant serological indexes of DN patients with hypertension. The pathogenesis of DN is complex and includes hemodynamic changes, metabolic disorders, inflammatory response, involvement of the kinin system and other factors. The pathological characteristics of DN are mainly renal microvascular injury, which can lead to high glomerular perfusion, increase the glomerular filtration rate, activate the renin angiotensin system, increase the blood pressure and aggravate the degree of renal injury.^9^ Colussi G and others^10^ showed that about 50% of patients with Type-2 diabetes had hypertension, and the incidence of hypertension in patients with Type-2 diabetes is 2-fold higher than in general population. FBG, systolic blood pressure and diastolic blood pressure are clinical indexes that are used to evaluate the effect of the treatment in DM patients with hypertension. Liu W and colleagues^11^ conducted a study on this group of patients treated with valsartan combined with nifedipine controlled-release tablets. It was found that FBG, systolic blood pressure and diastolic blood pressure decreased significantly after the treatment.

In agreement with these studies, our results indicated that while treatment with both regiments was associated with a significant decrease in the FBG, systolic and diastolic blood pressure, a combined treatment with valsartan and nifedipine controlled-release tablets resulted in significantly improved clinical indexes of hypertension as compared to treatment with nifedipine alone.

Numerous studies focused on identifying serological markers, such as COMP, ANG-1, Hcy, Tm, mALB, Cys C, TGF-β1, etc, that are associated with DN and hypertension. COMP is a member of the cysteine inhibition family. The study of Chen S et al.^12^ showed that the expression level of COMP was significantly upregulated in DN patients with hypertension in the study of the role of 12 new candidate nucleus pulposus (NP) markers in degenerative disc disease. ANG-1 is involved in angiogenesis and plays a role in the development of DN-related vascular disease. Yu J et al.^13^ found that angiotensin converting enzyme 2 (ACE2) and angiotensin (1-7) [Ang (1-7)] receptor axis may play a protective role in preventing myocardial remodeling in patients with hypertension. TM and mAlb are common markers of renal vascular injury and are often used in the evaluation of renal function. Hcy is a kind of sulfur-containing non-essential amino acid. Excessive Hcy deposition in blood can lead to excitotoxicity and plays a role in vascular endothelium injury by mediating oxidative stress response. CysC is a member of cysteine inhibitor family that can only be eliminated from the organism through kidneys. Together with Hcy, CysC promotes the progression of renal injury.^14^ TGF-β1 is a kind of active polypeptide, which can promote cell growth and differentiation and immune regulation and can induce renal fibrosis. The research of Zheng ZC et al.^15^ shows that TGF-β1 level is positively correlated with the progress of renal disease. APN is a specific index for the diagnosis of renal function. The renal clearance function can reduce the clearance and accumulation of APN. ALD can reflect the degree of vascular fibrosis. Ghrelin is a kind of adipokine, which can mediate angiogenesis and promote diabetic microangiopathy.^16^,^17^ The above indexes were selected as the serological indexes for result evaluation in this study. Our results showed that the levels of TM, mALB, Hcy, CysC, TNF-β1, ALD and ghrelin in the observation group were significantly lower than those in the control group (*P*<0.05), and the level of Ang-1 was higher than those in the control group (*P*<0.05). Our results suggest, therefore, that the combination of valsartan and nifedipine controlled-release tablets in the treatment of DN with hypertension facilitates better improvement the relevant serological indexes of patients as compared to nifedipine alone. Valsartan is a kind of angiotensin receptor antagonist, which can reduce blood pressure by inhibiting vasoconstriction. Nifedipine controlled-release tablet is a calcium channel antagonist, which can reduce blood pressure by relaxing arterial smooth muscle and expanding peripheral blood vessels. The combination of the two can play a synergistic role by reducing the vasoconstriction effect of angiotensin II and ɑ1 adrenoceptor, further improving the antihypertensive effect, delaying the progress of DN, and improving serological indexes.^18^,^19^

### Limitations of the study

This is a retrospective study with only 80 cases included. Additionally, there is no longer-term follow-up observation in combination with clinical treatment. These factors may result in a certain subjectivity, which may make the conclusions one-sided.

## CONCLUSION

The treatment of DN patients with hypertension with valsartan combined with nifedipine controlled-release tablets can further improve the curative effect and promote the improvement of relevant serological indexes. This study may provide some future references for the evaluation of the therapeutic effect of different treatment regimens in DN patients with hypertension.

### Authors’ contributions:

**LC:** Conceived and designed the study.

**LC & HZ:** Collected the data and performed the analysis.

**LC:** Was involved in the writing of the manuscript and is responsible for the integrity of the study.

All authors have read and approved the final manuscript.
